# Feasibility of an eHealth Service to Support Collaborative Depression Care: Results of a Pilot Study

**DOI:** 10.2196/jmir.1510

**Published:** 2010-12-19

**Authors:** Matic Meglic, Mirjana Furlan, Marja Kuzmanic, Dejan Kozel, Dusan Baraga, Irma Kuhar, Branko Kosir, Rade Iljaz, Brigita Novak Sarotar, Mojca Zvezdana Dernovsek, Andrej Marusic, Gunther Eysenbach, Andrej Brodnik

**Affiliations:** ^9^Centre for Global eHealth InnovationUniversity Health NetworkToronto, ONCanada; ^8^Department of Health Policy, Management and EvaluationUniversity of TorontoToronto, ONCanada; ^7^University Psychiatric Hospital LjubljanaLjubljanaSlovenia; ^6^Primary Health Care Centre BreziceBreziceSlovenia; ^5^Ambulanta Kosir d.o.o.Skofja LokaSlovenia; ^4^Psihiatricna Ambulanta VrhnikaVrhnikaSlovenia; ^3^Primary Health Care Centre CerknicaCerknicaSlovenia; ^2^Health Care Centre CeljenjeKoperSlovenia; ^1^Primorska Institute of Nature Science and TechnologyUniversity of PrimorskaKoperSlovenia

**Keywords:** Depression, patient care management, information systems, Internet, treatment outcome, medication adherence, pilot study, feasibility study, collaborative care

## Abstract

**Background:**

Treatments and organizational changes supported by eHealth are beginning to play an important role in improving disease treatment outcome and providing cost-efficient care management. “Improvehealth.eu” is a novel eHealth service to support the treatment of patients with depressive disorder. It offers active patient engagement and collaborative care management by combining Web- and mobile-based information and communication technology systems and access to care managers.

**Objectives:**

Our objective was to assess the feasibility of a novel eHealth service.

**Methods:**

The intervention—the “Improvehealth.eu” service—was explored in the course of a pilot study comparing two groups of patients receiving treatment as usual and treatment as usual with eHealth intervention. We compared patients’ medication adherence and outcome measures between both groups and additionally explored usage and overall perceptions of the intervention in intervention group.

**Results:**

The intervention was successfully implemented in a pilot with 46 patients, of whom 40 were female. Of the 46 patients, 25 received treatment as usual, and 21 received the intervention in addition to treatment as usual. A total of 55% (12/25) of patients in the former group and 45% (10/21) in the latter group finished the 6-month pilot. Available case analysis indicated an improvement of adherence in the intervention group (odds ratio [OR] = 10.0, *P* = .03). Intention-to-treat analysis indicated an improvement of outcome in the intervention group (ORs ranging from 0.35 to 18; *P* values ranging from .003 to .20), but confidence intervals were large due to small sample sizes. Average duration of use of the intervention was 107 days. The intervention was well received by 81% (17/21) of patients who reported feeling actively engaged, in control of their disease, and that they had access to a high level of information. In all, 33% (7/21) of the patients also described drawbacks of the intervention, mostly related to usability issues.

**Conclusions:**

The results of this pilot study indicate that the intervention was well accepted and helped the patients in the course of treatment. The results also suggest the potential of the intervention to improve both medication adherence and outcome measures of treatment, including reduction of depression severity and patients becoming “healthy.”

## Introduction

Depressive disorders are the second leading cause of disability worldwide with prevalence ranging from 16% to 18% during the entire life span [1]. The majority of people with depression are treated in primary health care [2,3]. It has been shown that treatment of depression in the primary care setting is far from optimal [4-6].

To improve the outcome of depression treatment, we need to improve patient adherence to therapy [7-9] and the care process itself using, for example, collaborative care, which is characterized by enhanced collaboration between the patient and health care professionals involved in the treatment process [10-12]. Glied has shown that particularly in collaborative care it appears possible to sustain net benefits using less costly interventions [10]. The question we asked was: Can we develop eHealth interventions to treat depression in which the net benefits are sustained while further reducing resource utilization and cost?

New eHealth tools and interventions promise to provide care process support (helping patients and health care professionals to comply with the defined care process with less effort) and to actively engage the patients, thus reducing resource usage [13,14]. Online self-treatment interventions have already proven their clinical value [15], and literature also describes eHealth solutions to support collaborative care in depression treatment [16] including eHealth solutions that have already demonstrated significant improvement of outcomes [17,18].

An eHealth system for active patient engagement and care management, called RecoveryRoad, has been described by Robertson [19]. Its features included secure e-consultations, progress-monitoring questionnaires, psycho-education, and evidence-based therapy. It also offered access to patients’ data, automated reminders for patients, and support for case management. In reports of preliminary findings, Robertson described high adherence to the system (53% to 84%) and self-reported medication adherence (over 90%) with a large effect size (Cohen’s *d* = 1.0) on average depression severity decline [19].

In this paper, we report the results of a pilot study to assess the feasibility of a novel eHealth intervention to support treatment of patients with depression.

## Methods

### Improvehealth.eu Intervention

The intervention, “Improvehealth.eu” service [20], consisted of (1) a Web-based information and communication technology system, referred to as “the ICT system,” designed to support collaborative care management and active patient engagement, and (2) online and phone-based care management performed by trained psychologists.

The intervention was administered via the Internet (accessible using personal computers and smart phones) and mobile phones. The ICT system, available 24/7, aimed to (1) actively engage the patients in the process of care; (2) increase the availability of information to all involved health care professionals (psychologists/care managers, general physicians, and psychiatrists); (3) automatically detect patient issues like poor or missing treatment response, unwanted side effects, emergence of suicidality, and nonadherence to medication regimens; and (4) provide timely response by care managers [21]. Care managers were available by telephone during service hours (3 hours per day on workdays), and their email response time was not longer than 2 working days. Upon starting the intervention, patients were informed that in case of imminent suicidality outside the intervention’s service hours they were to contact existing urgent psychiatric care providers. Care managers reported all patient-related activities in the ICT system, and physicians were asked to do the same.

Patients were actively engaged by submitting self-reporting questionnaires on symptoms and drug therapy side effects at least once per week in the acute phase (lasting from week 0 to week 9 after the start of therapy) and at least once per month in the continuation phase (lasting from week 10 to week 23). Submitted questionnaires provided real-time evaluation data for the ICT system and care managers.

The ICT system defined and assigned the tasks in the care process automatically for each patient. Some administrative and clinical tasks with well-defined trigger rules were performed by the ICT system in an automated way using rule sets and an evaluation matrix [21,22]. These tasks included (1) sending reminder text messages to patients and/or care managers if patients forgot to submit questionnaires on due dates, (2) quantitatively analyzing submitted questionnaires, and (3) creating questionnaire-related tasks for care managers (ie, to call a patient who had discontinued treatment).

The ICT system could only be accessed using secure hypertext transfer protocol (HTTPS) and digital certificates. All data were stored on the University of Primorska server in an encrypted file system. Access of health care professionals to data was only granted on patient consent.

Generic functionalities available to all users (patients, care managers, and care providers) included a personal calendar; an internal messaging system (a system of mailboxes); a forum as well as questions and answers; personal profile settings; information on depressive disorder, treatment, and emergency facilities; and the ICT system instructions.

**Figure 1:**
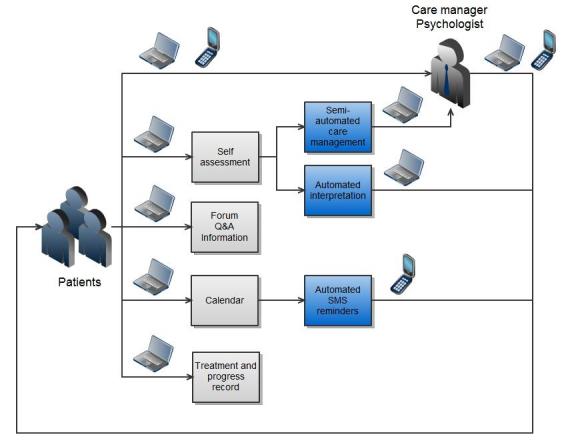
Simplified patient view of the intervention in which arrows describe the direction of information flow, boxes represent ICT system functionalities, and icons above arrows denote available channels (personal computer, mobile phone)

**Figure 2:**
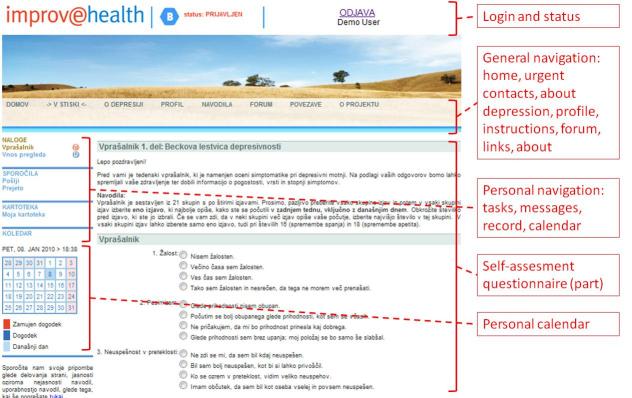
Screenshot of “Improvehealth.eu” in which a patient is submitting a questionnaire (the red semibrackets indicate particular modules, explained in boxes on the right hand side)

Additional functionalities of the ICT system were available to patients, care managers, and care providers. These are shown in Table 1. A schematic description of functionalities available to patients is given in Figure 1 (not all are shown). In addition, a screenshot of “Improvehealth.eu” in which a patient is submitting a questionnaire is depicted in Figure 2.

**Table 1:** Additional functionalities of the ICT system available to patients, care providers, and care managers

Additional Functionalities		Descriptions
**For patients**		
	Online self-assessment questionnaires (on depression symptoms, treatment side effects, suicidality, and medication adherence)	· composed of 46 questions over 2 pages with fixed question order · utilized adaptive questioning, that is, additional in-depth questions that appeared when certain answers were chosen · a completeness check upon each page submission · no review steps, that is, users could review the submitted questionnaire after submission in their record history but could not change it · available by using a link on the homepage · information from all completely submitted questionnaires used in the analyses
	Automated personalized interpretations	· provided to the user by the ICT system instantly after the online questionnaire submission · included a tailored reinforcement message · provided analysis of deviations such as lack of symptom improvement or emergence of side effects
	Access to a psychologist/care manager	· available over internal messaging and phone during predefined hours · no psychotherapy offered apart from unstructured conversations (in contrast to RecoveryRoad [19])
	Automated text message reminders	· sent to patients’ mobile phones in case of overdue tasks such as booking an appointment with their physicians or submitting a questionnaire
	An individual patient record	· included submitted questionnaires and reports of patient-professional interaction by care managers and physicians sorted by time · internal to the ICT system · by clicking on a particular entry, the entry would expand to show all stored information
**For care managers and care providers**		
	Dashboard	· provided a patient list with status indicators (symptoms, suicidality, medication adherence, etc) · included task lists for at-a-glance overview
	Semi-automated care management	· triggered by an automated analysis of each self-assessment questionnaire upon which specific tasks were automatically assigned to care managers by the ICT system
	Activity forms for reporting of performed tasks	· supported monitoring of timely execution · different forms for different professionals
	An e-learning module:	· provided the latest treatment guidelines and a related online test for physicians to earn continuing medical education points

### Study Design

The pilot study to explore the feasibility of the intervention was approved by the Slovene National Medical Ethics Committee. Inclusion criteria were: a diagnosis of depression (ICD10 group F32) or mixed anxiety and depression disorder (ICD10 code F41.2) for the first time or after a remission of at least 6 months; introduction of antidepressant treatment in the last 10 days; regular use of Internet and mobile phone; 14 or more points on Beck Depression Inventory-II (BDI-II) questionnaire [23,24].

The control group received treatment as usual, that is, physician visits and antidepressant treatment. The intervention group received the intervention “Improvehealth.eu” service as an addition to treatment as usual. Systematic alternating order (unweighted even-odd distribution) without blinding was used to assign patients to the control group or to the intervention group [25].

After giving informed consent, the patients filled in 2 paper questionnaires, one at the beginning of the pilot (referred to as Time 0) and the other after 24 weeks, that is, at the end of the pilot (referred to as Time 1). The Time 0 assessment consisted of a questionnaire assessing demographics and BDI-II [23,24]. The Time 1 assessment consisted of a repeated BDI-II assessment and a 26-question questionnaire exploring the duration of antidepressant therapy, side effects, adherence, patients’ perceived quality of care, and, for the intervention group only, the overall perception of the intervention.

Patients were recruited and enrolled by 7 physicians upon initial assessment. The observation period for each individual patient was 6 months. Primary outcome measures were patients’ medication adherence and clinical outcome measures (reduction in depression severity according to BDI-II and reaching “healthy” criteria, described below). User acceptance and usage patterns were explored as secondary outcome measures. No incentives were offered to patients for finishing the pilot.

#### Demographics and Pilot Participation

Demographics included age, gender, marital status, education, and employment. Attrition rate was measured as the share of patients responding to the Time 1 questionnaire.

#### Medication Adherence, BDI-II Improvement, and Outcome Measures

For self-assessment of medication adherence at Time 1, we used a questionnaire combining 3 previously reported measures: (1) regularity of administration over the defined medication period, (2) taking the medication at the same time of the day, and (3) regular use of correct dosage [26-28]. “Adherent” was defined as adherent to 2 or 3 of these criteria.

To assess reduction in depression severity, we calculated the difference between patient-reported Beck Depression Inventory-II (BDI-II) scores at Time 0 and Time 1 [23,24]. To assess clinically important change (the patient becoming “healthy”), we used a combination of BDI-II score of less than 14 points at Time 1 and at least 8 points improvement in BDI-II score from Time 0 to Time 1 as suggested in previous research (rounded from 14.29 and 8.46, respectively) [29-31].

#### Usage Patterns

Additional data were acquired from the database to explore the duration and frequency of intervention usage and workload on care managers, including time between registration and last submitted questionnaire, number of submitted questionnaires, and number of tasks performed by care managers.

#### Patient Feedback

For qualitative assessment, the patient questionnaires at Time 1 included open-ended questions on overall satisfaction with the intervention. They also included 12 Likert-type items (statements) on patient perception of care quality, access to care, and access to information.

#### User Experience With the ICT System

Initial usability testing of the ICT system was performed before the pilot by 6 healthy individuals and 1 usability expert. Users had to perform tasks like registering, filling in the questionnaire etc. Usability issues regarding the ICT system arising during the pilot were reported and listed as such.

### Statistical Analysis

Choice of tests was dependant on variable type. Two-sided significance testing was used in all cases. To compare demographic characteristics of the two groups we used the Fisher exact test, the Mann-Whitney test, and the chi-square test. For adherence, Mantel-Haenszel odds ratio estimate was used. For BDI-II improvements, we performed available case analysis using paired and unpaired *t* tests and Cohen’s *d* for effect size. For outcome measures, we used the Fisher Exact test and odds ratio estimates.

We employed a simple sensitivity analysis to assess variability due to dropouts’ missing data: available case and intention-to-treat analyses were performed, the latter using simple imputation scenarios [32]. These scenarios were: (1) “healthy” (ie, an assumed BDI-II score of less than 14 and symptom reduction of at least 8) early quitters in the intervention group were also healthy at Time 1, and the average frequency of healthy dropouts in the control group was the same as in the intervention group; (2) all drop-outs in either group were not healthy; or (3) all dropouts in both groups were healthy. These 3 scenarios imputed the same risks in both groups, pulling effect estimates towards the null hypothesis [32], thus avoiding the overestimation of intervention effect. We also added a pessimistic scenario (4) in which all missing patients in the intervention group were imputed as “not healthy,” whereas in the control group they were imputed as “healthy.”

Usage patterns were assessed using Kaplan-Meyer analysis. The 2 scenarios used were (1) treating all patients as events and (2) treating “healthy” quitters (patients quitting with last reported BDI-II values reaching “healthy” criteria) as censored events. Care manager usage patterns were listed depending on classification of tasks.

For patient feedback, the qualitative answers were categorized and compared with the aims of the intervention, whereas Likert-type items were analyzed using the Mann-Whitney test.

## Results

### Demographics and Pilot Participation

There were no significant differences between the intervention and control groups at the beginning of pilot (Time 0 in Table 2) and after 6 months (Time 1) for age, gender, marital status, education, and employment. Of the 46 patients, 25 (54%) were allocated to control group and 21 (46%) to intervention group. The response rate at 6 months (Time 1) was slightly higher in the intervention group (12 out of 21, 57%) versus the control group (10 out of 25, 40%), but the difference was not statistically significant (χ^2^_1_ = 1.34, *P* = .38).

In the intervention group, the reasons patients gave for dropping out were dissatisfaction with the intervention (1 patient) and early significant clinical improvement (8 patients). For those who said they dropped out because they were much improved, it is unknown whether they were still healthy at Time 1. Reasons for high attrition at Time 1 in the control group were not known.

**Table 2:** Group characteristics at Time 0

		Intervention	Control	*P* value^a^
		n = 21	n = 25	
Age, median (mean ± SD)		36 (35.71 ± 12.11)	37 (40.04 ± 17.07)	.44^b^
Female gender, n (%)		18 (86%)	22 (88%)	.99^c^
**BDI-II symptom severity**				
	Mild, n (%)	2 (10%)	4 (16%)	
	Moderate, n (%)	8 (38%)	9 (36%)	
	Severe, n (%)	11 (52%)	12 (48%)	.81^d^
Married or partnered, n (%)		15 (71%)	17 (68%)	.99^c^
University degree, n (%)		8 (38%)	7 (28%)	.53^c^
Currently employed, n (%)		15 (71%)	12 (48%)	.14^c^

^a^ Comparison of the intervention group and the control group

^b^ Mann-Whitney test

^c^ Fisher exact test

^d^ Chi-square test

### Medication Adherence, BDI-II Improvement, and Outcome Measures

In the control group, 3 out of 9 (33%) patients were adherent to antidepressants compared with 10 out of 12 patients (83%) in the intervention group (χ^2^_1_ = 5.45, *P* = .03, odds ratio [OR] = 10.0, 95% confidence interval [CI] = 1.28-78.1).

Both groups demonstrated significant within-group reduction of mean BDI-II score from Time 0 to Time 1 (control group: paired *t*_9_ = 3.95, *P* = .003, Cohen’s *d* = 1.23; intervention group: paired *t*_11_ = 7.23, *P* < .001, Cohen’s *d* = 2.57), with intervention group seeming to indicate a greater effect size, which is further supported by the between-group comparison shown in Table 3.

**Table 3:** Between-group comparison of BDI-II for available cases

	Intervention Mean (SD)	Control Mean (SD)	Difference	Two-sample t_20_; *P* value	Effect Size: Cohen’s *d* (95% CI)
	n = 12	n = 10			
BDI-II at Time 0	29.50 (8.15)	28.70 (8.34)	-0.80	0.23; *P* = .82	
BDI-II at Time 1	9.83 (8.05)	17.80 (7.91)	7.97	2.33; *P* = .03	1.00 (0.09-1.88)

In outcome sensitivity analysis (Table 4), available case analysis and the 3 intention-to-treat (ITT) scenarios with equal risk imputation [32] resulted in odds ratios in favour of intervention, seeming to indicate an improvement of outcome in the intervention group. The last, pessimistic scenario was insignificantly in favour of the control group. Confidence intervals were wide due to small sample sizes and high dropout ratios.

**Table 4:** Outcome measures: available cases and intention-to-treat (ITT) analysis scenarios

		Intervention	Control	χ^2^_1_, *P* Value	Odds Ratio (95% CI)
Healthy at Time 0, n (%)		0/21 (0%)	0/25 (0%)		
Healthy at Time 1, n/available cases (%)		9/12 (75%)	1/10 (10%)	9.3, *P* = .004	27 (2.3-310)
**Intention-to-treat scenarios**					
	Healthy at Time 1, n (%): ITT 1^a^	17/21 (81%)	15/25 (60%)	2.4, *P* = .20	2.8 (0.73-11)
	Healthy at Time 1, n (%): ITT 2^b^	18/21 (86%)	17/25 (68%)	2.0, *P* = .19	2.8 (0.64-12)
	Healthy at Time 1 (%): ITT 3^c^	9/21 (43%)	1/25 (4%)	10.1, *P* = .003	18 (2.0-159)
	Healthy at Time 1 (%): ITT 4^d^	9/21 (43%)	13/25 (68%)	2.9, *P* = .14	0.35 (0.12-1.2)

^a^ realistic in that “healthy” early quitters in the intervention group were healthy at Time 1, and the average frequency of healthy dropouts in the control group was the same as in the intervention group

^b^ all missing patients from either group are assumed “healthy”

^c^ all missing patients from either group are assumed “not healthy”

^d^ pessimistic in that all missing in the intervention group assumed “not healthy” and all missing in control group assumed “healthy”

### Usage Patterns

**Figure 3:**
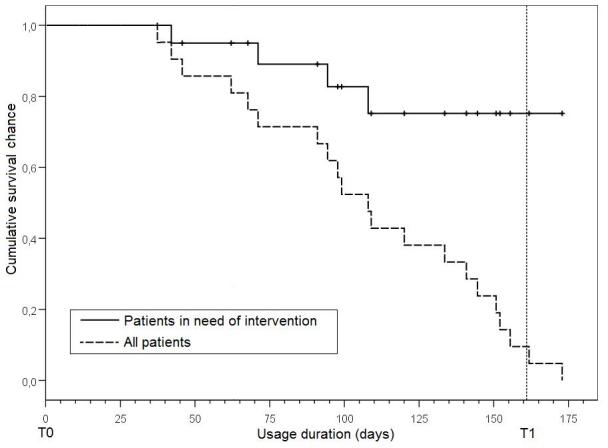
Kaplan-Meier survival analysis for use of intervention (dotted vertical line denotes Time 1 at beginning of the 24th week)

In Figure 3, the Kaplan-Meyer plot depicts the chance of a patient reaching a certain duration of intervention usage. Shown are 2 scenarios: (1) all patients and (2) only patients in need of further intervention where only nonhealthy patients at any given time were taken into account (by treating healthy quitters as censored events). The mean duration of intervention usage by all patients was 107 days (95% CI 90-125 days); for patients in need of further intervention the mean duration was 150 days (95% CI 131-170 days).

The patients submitted a total of 431 questionnaires online of which 198 (46%) were complete. The average number of complete questionnaires per patient was 9.9 (SD 3.35, range 3-14). The remaining 229 submitted questionnaires were only partially completed and were treated as unsuccessful submissions. The patients were required to fill in the missing answers and resubmit the questionnaires.

Of the 21 patients in the intervention group, 6 (29%) required guided registration over the phone by the care manager. Care managers submitted 46 task-resolution reports related to 16 of the 21 patients (76%) (see Table 5).

**Table 5:** Care manager tasks

Reason	Number of Tasks	% of Tasks	Patients Involved, n (%) (n = 21)^a^
Questionnaire overdue: phone patient	15	33%	9 (43%)
Reported side effects of medication: phone patient	6	13%	2 (10%)
Reported suicidality: phone patient	3	7%	2 (10%)
Confirm change of therapy: contact physician	2	4%	2 (10%)
Missing symptoms improvement: phone patient	5	11%	5 (24%)
Due date of control visit: phone patient	9	20%	6 (29%)
Exit/dropout: phone patient	6	13%	3 (14%)
**Total**	**46**	**100%**	**16^a^ (76%)^b^**

^a^ The number of total patients involved in task resolution (16) is less than the number of patients in the intervention group (21) as for some patients no tasks were assigned to the care manager.

^b^ As some patients required that care managers undertake tasks for more than one reason, the sum of total patients involved is less than the sum of involved patients by reasons.

In addition, care managers reported that 33 assigned tasks were not resolved because the patient did not answer the phone or reply to an email. Of these, 88% (29/33) were requested for patients who dropped out. The average number of tasks performed was 2.2 per patient and 2.9 per patient actually requiring that the case manager undertake a task. In addition, 1 of the 7 physicians involved in the pilot reported patient visits in the ICT system, and none of the 7 physicians performed the e-learning test.

### Patient Feedback

No significant differences were detected between the control group and the intervention group in perception of care quality or accessibility to care and information. Qualitative feedback regarding the intervention provided by patients from the intervention group is shown in Tables 6. Of the 21 patients, 17 (81%) gave positive feedback whereas 7 (33%) gave negative feedback.

**Table 6:** Positive feedback provided by patients in the intervention group: categories and examples

Category of Intervention Benefit	Number of Replies (n = 17)	Example
Increased control of their disease and improved overview	6 (35%)	I could monitor my progression.
Provided an incentive	3 (18%)	It was reminding me of regular antidepressant intake.
Useful information, increased knowledge	2 (12%)	Improved knowledge of depression and how to fight it.
Available and responsive	2 (12%)	Quick coordination, quick advice, quick transfer of information.
Treatment barrier reduction	2 (12%)	Much easier to communicate over the internet than live.
Overall usefulness	2 (12%)	I liked everything.
			

**Table 7:** Negative feedback provided by patients in the intervention group: categories and examples

Category of Intervention Drawback	Number of Replies (n = 7)	Example
Annoying	2 (29%)	Annoying text messages
Repetitive	2 (29%)	Same questionnaire repeating all the time
Computer literacy required	2 (29%)	Some computer literacy is needed; digital certificate installation difficulty
Lack of content	1 (13%)	Empty forum, empty question and answer

### User Experience With the ICT System

Some areas for improvement were identified during the pilot. The following 4 required increased resource utilization and called for a future modification of work processes: (1) Digital certificates (electronic documents required by the ICT system from each user for authentication) required time and were somewhat difficult for both patients and care managers to manage. Further simplifications of certificate handling are necessary and human resources are required to help patients register. In the future, we anticipate that digital certificate “literacy” among users will reduce the importance of this issue. (2) A significant proportion of the care manager workload was due to dropouts not responding to calls and emails. More efficient strategies for interaction with these patients are needed. (3) Physician usage of the ICT system was poor, requiring specific motivational strategies (ie, a reimbursement scheme). (4) Frequently asked questions were available but not used, as the protocol that required the care manager to post these was not strictly enforced.

An additional 4 areas were identified that require changes in the ICT system functionality: (1) The feedback provided when a patient does not complete a questionnaire needs to be improved (ie, that directs users to missing answers). (2) Automated text messages were seen as a disturbance for a minority of patients who experienced fast clinical improvement and wished to finish the intervention early. The solution is for the care manager to have the ability to deactivate these messages for individual patients. (3) The knowledge base of the ICT system needs to be upgraded to increase the pool of available interpretations when questionnaires are submitted. The reason for this is to reduce repetitive answers that are possibly perceived as impersonal by patients. (4) Because the forum was poorly used and the clinical value of forums is unclear [33,34], consideration should be given to discontinuing of this function.

## Discussion

The main findings were that (1) user feedback confirmed the ICT system’s alignment with the initial objectives (active patient engagement and improved care management) and (2) the results of the pilot indicate the intervention’s likely influence on improvement of medication adherence and the outcome measures, namely the reduction of depression severity and patients becoming “healthy.”

Overall usability was good, with some feature enhancements necessary to further improve it. Patient feedback about the benefits of the ICT system was in line with the intervention goal and its design. The intervention seemed to support collaborative care [16,35] and active engagement [36] if usability issues are addressed properly in future [37].

We noticed that medication adherence for treatment as usual was low (33%) and comparable to values reported previously (21%) [38]. The intervention group had a significantly higher adherence rate (83%), likely due to the intervention.

The effect size of improvements in scores on the BDI-II in the intervention group compared with the control group was in line with the results of the study by Robertson et al [19] (in both cases Cohen’s *d* = 1). This seems to indicate an effect of the intervention on treatment success and is further supported by improved outcome measures for intervention group. No significant changes in treatment quality perception were observed.

### Limitations

Even though we employed tests that gave the highest statistical power for a given variable type (parametric where applicable, nonparametric otherwise), small sample sizes in most analyses and the additionally high dropout ratio for intention-to-treat analyses resulted in wide confidence intervals. The fixed allocation sequence with even-odd randomization and no blinding [25] likely contributed to bias, and other contributing factors such as depression severity and comorbidities were also not taken into account. We suggest larger sample sizes and more robust methodology (with improved dropout prevention, full randomization, and use of advanced imputation techniques) for further research of the topic.

### Conclusions

This pilot study has shown that the intervention—a novel eHealth service offering collaborative care management and active patient engagement—was well received by potential users, seeming to indicate increased patient engagement and feelings of control over treatment progress. The pilot also seems to indicate a likely positive effect of this type of intervention on medication adherence and outcome measures in depression treatment, possibly further improving outcome in addition to interventions offering online cognitive behavioral therapy [39-41].

## Abbreviations

HTTPS: secure hypertext transfer protocol
ICT: information and communication technology
ITT: intention-to-treat
BDI-II: Beck Depression Inventory-II
